# G protein-coupled receptor-mediated renal fibrosis: a key focus on kidney disease drug development

**DOI:** 10.3389/fphar.2025.1645888

**Published:** 2025-09-04

**Authors:** Hui Wang, Mengfan Yang, Xiongfeng Liu, Junming Fan, Can Wang

**Affiliations:** ^1^ Institute of Herbgenomics, Chengdu University of Traditional Chinese Medicine, Chengdu, China; ^2^ School of Clinical Medicine, Chengdu University of Traditional Chinese Medicine, Chengdu, China; ^3^ Department of Nephrology, First Affiliated Hospital of Chengdu Medical College, Chengdu, China

**Keywords:** GPCRs, renal fibrosis, signal transduction, drug development, phenotypic transformation

## Abstract

Renal fibrosis (RF) represents the pathognomonic end-stage phenotype of progressive nephropathies, pathologically characterized by excessive deposition of fibrillar extracellular matrix (ECM) and irreversible obliteration of parenchymal architecture. G protein-coupled receptors (GPCRs)—members of the heptahelical transmembrane receptor superfamily—function as master regulators orchestrating both physiological renal homeostasis and maladaptive fibrotic reprogramming in response to injury. Despite robust clinical evidence validating the therapeutic tractability of GPCR-targeted interventions for chronic kidney disease (CKD), no approved agents specifically antagonize the core pathogenic drivers of RF. Consequently, this review systematically delineates GPCRs exhibiting mechanistic primacy in RF pathobiology and translational promise, with focused interrogation of endothelin receptors, angiotensin receptors, chemokine receptors, and adenosine receptors. Beyond canonical modulation of inflammatory leukocyte infiltration and pro-fibrotic phenotypic transitions, emerging paradigms highlight GPCR governance over metabolomic reprogramming and mechanotransductive signaling during fibrogenesis. Notwithstanding these mechanistic advances, clinical translation of GPCR-directed anti-fibrotic therapeutics remains nascent, constrained by target pleiotropy, biodistribution barriers, and species-divergent pathophysiology. Collectively, GPCRs constitute high-value molecular targets for intercepting the progression of RF at its mechanistic nexus.

## Graphical Abstract

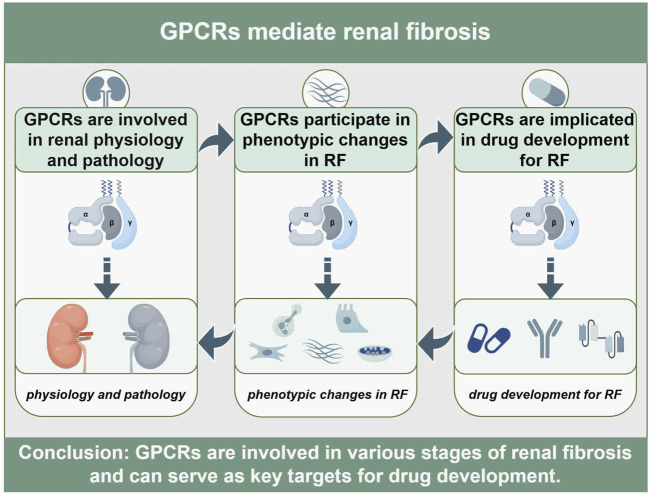

## 1 Global challenge of renal fibrosis

Since 1990, the global burden of chronic kidney disease (CKD) has escalated markedly, with prevalence increasing by 29.3%, and mortality rising by 41.5%, constituting a major public health challenge (GBD Chronic Kidney Disease, 2020). However, early-to-moderate stage CKD is highly preventable and potentially reversible (Shlipak et al., 2021). Regrettably, the clinically silent nature of incipient CKD precludes timely intervention, frequently permitting inexorable progression to end-stage renal disease (ESRD). This trajectory is evidenced by a doubling of ESRD prevalence over the past 2 decades (Kuehn, 2022). Renal fibrosis (RF) represents the terminal pathological convergence in CKD progression, morphologically characterized by glomerulosclerosis, tubular atrophy, vascular rarefaction, and interstitial fibrosis, culminating in excessive extracellular matrix (ECM) deposition and scar formation (Huang et al., 2023; Li L. et al., 2022). Conventionally, RF pathogenesis was attributed to aberrant cellular phenotypic plasticity, encompassing mesenchymal transformation of renal epithelial and endothelial cells (EMT/EndMT) and pathological activation of matrix-producing myofibroblasts (Yamashita and Kramann, 2024). However, contemporary research has elucidated previously unrecognized regulatory axes governing fibrogenic commitment, including non-coding RNA networks (Van der Hauwaert et al., 2019), epigenetic modifications (Li X. et al., 2022), metabolic reprogramming (Zhu et al., 2021), and extracellular vesicles-mediated signaling (Kosanović et al., 2021). These mechanisms present promising direction for modulating—and potentially reversing—established fibrosis. Despite these mechanistic advances, no therapeutics directly and selectively targeting RF pathogenesis are clinically available (Huang et al., 2024). Consequently, current clinical practice relies on agents developed for broader CKD management—angiotensin-converting enzyme inhibitors (ACEIs), angiotensin II receptor blockers (ARBs), mineralocorticoid receptor antagonists (MRAs), and sodium-glucose co-transporter 2 inhibitors (SGLT2i) —to indirectly attenuate fibrotic progression (Wang and Zhang, 2024). Nevertheless, their efficacy remains suboptimal and variable, while safety profiles are constrained by underlying etiological heterogeneity, disease stage disparities, and diverse environmental determinants (Reiss et al., 2024).

Consequently, our focus centers on the G protein-coupled receptors (GPCRs) superfamily, Representing the largest cohort of human membrane proteins and historically constituting the most therapeutically exploited target class, GPCRs hold profound significance (Zhang et al., 2024). Within nephrology, GPCR-directed pharmacotherapies have established pivotal clinical utility (Lv et al., 2024; Tang et al., 2025). The most substantiated classes encompass AT1R antagonists (Rianto et al., 2021), GLP-1R agonists (Rossing et al., 2023), ETR antagonists (Martínez-Díaz et al., 2023) and dual angiotensin/endothelin receptor antagonists (Kohan et al., 2024), collectively demonstrating immense promise for innovative renal disease drug development (Table 1). Critically, GPCR-targeted agents constitute the predominant share of receptor-focused therapeutic candidates in current clinical trials for RF (Abbad et al., 2025). Moreover, we emphasize that GPCR signal transduction and functionality are intimately implicated in the initiation and modulation of RF (Tang et al., 2025). Consequently, despite the formidable global challenge of developing effective clinical interventions for RF, the therapeutic promise of targeting GPCRs—leveraging their well-defined pathophysiological roles and notable inherent druggability—is increasingly commanding significant scientific and clinical attention.

**TABLE 1:** Clinical trial drugs targeting GPCRs for CKD.

GPCRs	Drug	Mechanism	Diseases	Outcomes	Ref.
ETAR	Atrasentan	Antagonist	T2D	Reduction in the risk of renal events	Heerspink et al. (2019)
	Zibotentan	Antagonist	CKD	High-dose zibotentan elevates fluid retention risk, whereas low-dose zibotentan combined with dapagliflozin mitigates this adverse effect	Smeijer et al. (2024)
ETAR/ETBR	Bosentan	Antagonist	T2D and microalbuminuria	Improvement of peripheral endothelial function in type 2 diabetic patients with microalbuminuria	Rafnsson et al. (2012)
AT1R	Irbesartan	Antagonist	DN	Reduction of proteinuria and attenuation of progression to ESRD	Ros-Ruiz et al. (2012)
	losartan	Antagonist	DN	iminution of urinary protein excretion with concomitant preservation of renal function	Brenner et al. (2001)
	Olmesartan	Antagonist	CKD and hypertensive patients	Reduction in nighttime BP with concomitant renal injury inhibition	Yanagi et al. (2013)
	Telmisartan	Antagonist	CKD	Decrease in urinary protein levels	Nakamura et al. (2010)
AT1R/ETAR	Sparsentan	Antagonist	IgAN	Amelioration of proteinuria and maintenance of kidney function	Rovin et al. (2023)
GLP1R	Dulaglutide	Agonist	T2D	Decrease in composite renal endpoint incidence	Gerstein et al. (2019)
	Liraglutide	Agonist	T2D	Nephroprotective effects, particularly in individuals with prior chronic kidney disease	Shaman et al. (2022)
P2RY12	Prasugrel	Antagonist	CKD	Suppression of platelet reactivity	Melloni et al. (2016)
	Ticagrelor	Antagonist	CKD	Mitigation of hemorrhagic risk	Stefanini et al. (2021)
CASR	Cinacalcet	Agonist	hemodialysis with moderate to severe secondary hyperparathyroidism	Suppression of serum PTH concentrations	Block et al. (2017)
KOR	Difelikefalin	Agonist	non-dialysis-dependent CKD and those undergoing hemodialysis	Attenuation of itch intensity	Yosipovitch et al. (2023)
HRH1	Fexofenadine	Antagonist	DN	Reduction in UACR	El-Fatatry et al. (2024)
PE2R1	Iloprost	Agonist	contrast-induced nephropathy	Protection against contrast-induced nephropathy in high-risk patients undergoing coronary procedures	Spargias et al. (2009)
CYSLTR1	Montelukast	Antagonist	Uremic Pruritus	Alleviation of uremic pruritus	Hercz et al. (2020)
A2AR	Pentoxifylline	Antagonist	DN	Reduction of albuminuria and conservation of residual eGFR	Navarro-González et al. (2015)
V2R	Tolvaptan	Antagonist	ADPKD	Deceleration of renal enlargement and functional decline	Torres et al. (2012)

Clinical Trial Drugs Targeting GPCRs, for CKD., T2D: Type 2 Diabetes; DN: diabetic nephropathy; IgAN: IgA nephropathy; ESRD: end-stage renal disease; PTH: parathyroid hormone; UACR: urinary albumin-to-creatinine ratio; eGFR: estimated Glomerular Filtration Rate; ADPKD: autosomal dominant polycystic kidney disease.

## 2 GPCR signaling transduction paradigms and targeted modulation strategies

**FIGURE 1:**
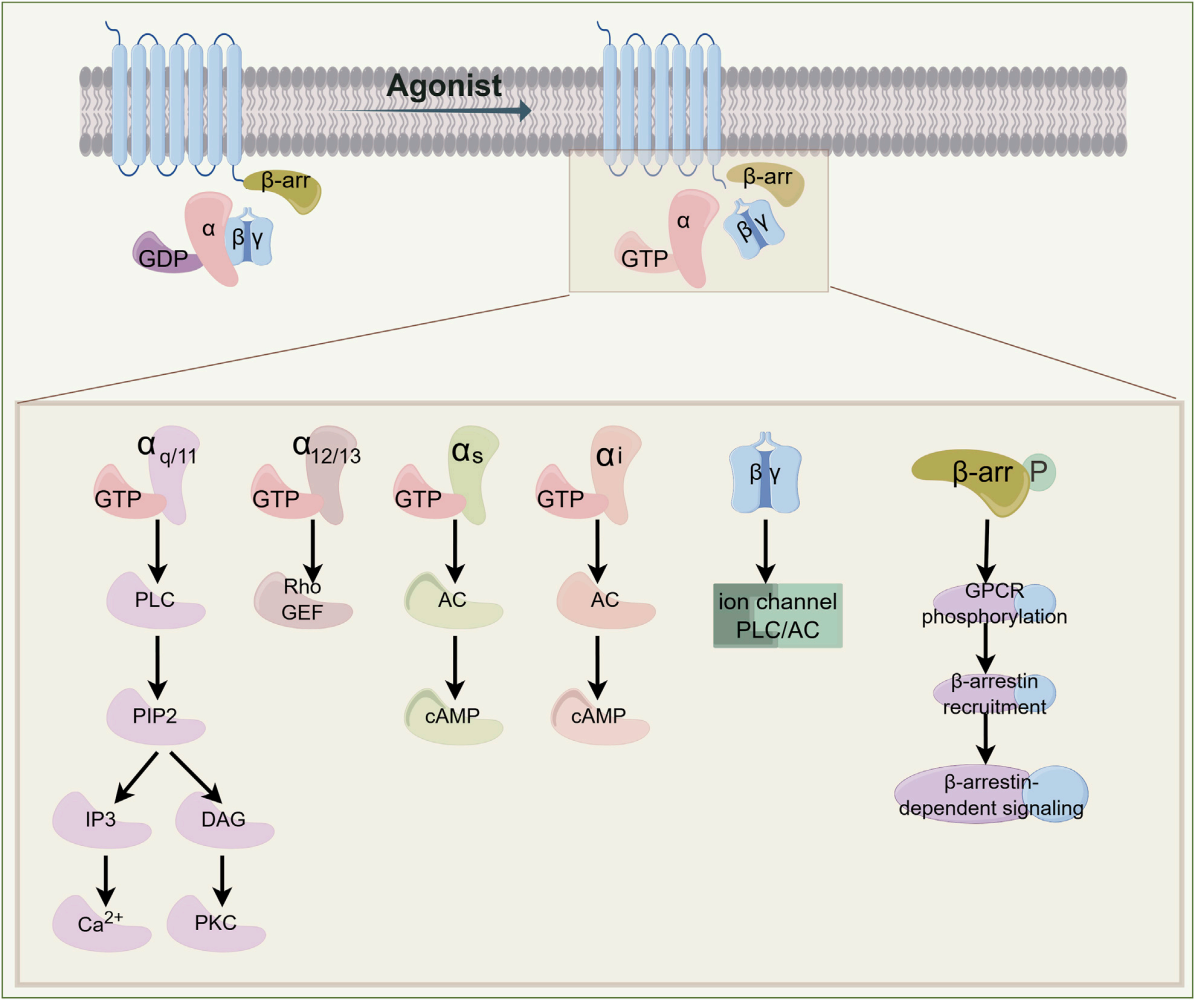
Signal Transduction Paradigm of GPCR. Ligand-receptor binding induces conformational changes in G proteins, triggering receptor activation. Different G protein subunits regulate distinct effector enzymes or ion channels, generating second messengers such as Ca^2+^, cAMP, and IP3. These signaling cascades initiate cellular responses, whereas receptor desensitization leads to signal termination. β-arrestin signaling is involved in receptor desensitization and endocytosis. GPCR: G-protein-coupled receptor; GTP: guanosine triphosphate; GDP: guanosine diphosphate; PLC: phospholipase C; PIP2: phosphatidylinositol 4,5-bisphosphate; IP3: inositol trisphosphate; DAG: diacylglycerol; PKC: protein kinase C; RhoGEF: Rho guanine nucleotide exchange factor; AC: adenylyl cyclase; cAMP: cyclic adenosine monophosphate.

GPCRs belong to the family of seven-transmembrane proteins. The human genome encodes approximately 800 GPCRs that orchestrate diverse physiological and pathophysiological processes across multiple organ systems (Congreve et al., 2020). The transmembrane helix structure comprises the extracellular N-terminus, three extracellular loops, an intracellular C-terminus, and three intracellular loops. Heterotrimeric G proteins, consisting of α, β, and γ subunits, serve as primary signaling partners. In the basal state, Gα subunits remain guanosine diphosphate (GDP)-bound and conformationally constrained. Ligand engagement induces allosteric transitions within the receptor’s transmembrane core, catalyzing GDP-guanosine triphosphate (GTP) exchange on the Gα subunit, This nucleotide switch triggers dissociation of the GTP-bound Gα subunit from the Gβγ dimer (Ballante et al., 2021) (Figure 1). The liberated Gα-GTP complex and Gβγ heterodimer regulate distinct downstream effectors. Gα subunits are phylogenetically categorized into four classes: Gαs, Gαi/o, Gαq/11, and Gα12/13. For example, Gαs primarily activates adenylate cyclase (AC), promoting the production of cAMP. Conversely, Gαi/o inhibits AC and cAMP activity; Gαq/11 binds with phospholipase C-β (PLCβ) to promote the hydrolysis of phosphatidylinositol-4,5-bisphosphate (PIP2) into inositol 1,4,5-trisphosphate (IP3) and diacylglycerol (DAG), which further activates downstream protein kinase C (PKC) and triggers Ca^2+^ release. The downstream signaling of Gα12/13 primarily involves Rho GTPase, with a more complex and diverse regulatory pattern (Rasheed et al., 2022; Jiang et al., 2022). The Gβγ complex independently regulates ion channels, kinases, and secondary messenger systems (Senarath et al., 2018). Signal termination is mediated by regulator of G protein signaling (RGS) domains, which accelerate GTP hydrolysis via intrinsic GTPase-activating protein (GAP) activity. Gα-GDP subsequently reassociates with Gβγ, reconstituting the inactive heterotrimer and completing the catalytic cycle (Masuho et al., 2023). Additionally, GPCR activation is partially independent of G proteins. For example, phosphorylated GPCRs recruit β-arrestins, which prevent G protein signaling and promote receptor internalization, initiating new signaling pathways (Asher et al., 2022). Furthermore, most adhesion GPCRs (aGPCRs) contain a special domain with a hydrolysis site. Their self-proteolysis leads to aGPCR autoactivation, causing the separation of Gα from Gβγ and initiating downstream signaling (Zhu X. et al., 2022).

Concurrently, emerging research has revealed intimate connections between RF pathogenesis and GPCR signaling cascades. Therapeutic targeting of the cAMP/PKA pathway (Stokman et al., 2021), Gβγ-GRK2 interface (Rudomanova and Blaxall, 2017), and β-arrestin-dependent signaling (Gu et al., 2015) has emerged as a validated strategy for antagonizing RF progression. Furthermore, the intricate crosstalk among non-coding RNAs (ncRNAs), epigenetic modifications, and GPCR regulation constitutes a pivotal investigative frontier (Zhao et al., 2014; Alghamdi et al., 2018; Liu et al., 2019). Supporting this paradigm, transcriptomic profiling of proximal tubule-mediated RF identifies 143 differentially expressed lncRNAs and 91 dysregulated GPCRs (Wu H. et al., 2020), whereas CaSR signaling—primarily orchestrating Ca^2+^ and water transport—demonstrates extensive miRNA interactions (Ranieri, 2019). Critically, bidirectional regulatory crosstalk exists between GPCR signaling cascades and ncRNAs networks. GLP-1R not only governs the circ8411/miR-23a-5p axis to mitigate lipid toxicity and endothelial pyroptosis (Wu W. et al., 2024) but is reciprocally modulated by extracellular vesicle-encapsulated miR-192 to exert renoprotection (Jia et al., 2018). Moreover, In butyrate-mediated protection against diabetic nephropathy (DN), GPR41, GPR43, and GPR109A engage in crosstalk networks involving histone deacetylase (HDAC) inhibition, histone butyrylation, and miRNA repertoire alterations, collectively modulating DN-associated inflammatory and fibrotic pathologies (Cheng et al., 2022). Notably, GPR109a activation rectifies promoter region acetylation and methylation patterns, preserving glomerular basement membrane (GBM) integrity (Felizardo et al., 2019). These findings establish ncRNAs and epigenetic machinery as critical upstream regulators of GPCR functionality, thereby revealing their therapeutic potential as precision targets for renal fibrosis intervention.

## 3 GPCRs are involved in regulating renal physiology and pathology

Within the kidneys, GPCRs exhibit ubiquitous expression and critically orchestrate essential physiological processes including renal development, fluid-electrolyte homeostasis, and blood pressure regulation (Table 2). Spatiotemporal mapping of GPCR distribution across nephron segments reveals prominent enrichment of aGPCRs, adrenergic receptors (ARs), and lysophosphatidic acid receptors (LPARs) along renal tubules (Poll et al., 2021). In alignment with prior evidence of olfactory receptors (ORs) participating in renal physiology (Kalbe et al., 2016), this profiling further identifies substantial enrichment of ORs along the nephron (Poll et al., 2021). In the renal vasculature and glomeruli, receptors including GPR91, GPR43, and apelin receptor (APJ) are functionally co-expressed and collectively participate in renal physiological regulation (Rajkumar and Pluznick, 2017). Moreover, Transcriptomic profiling identifies 56 GPCRs dysregulated in activated renal fibroblasts, underscoring their pathogenic involvement in fibrogenesis (Kaur et al., 2023). Developmental regulation is exemplified by GPR126, which exhibits progressive upregulation in ureteric buds and renal epithelia during murine nephrogenesis. Its persistent expression in mature tubular epithelium and collecting ducts implicates roles in progenitor cell differentiation and renal morphogenesis (Cazorla-Vázquez and Engel, 2018; Cazorla-Vázquez et al., 2023). Apically expressed GPR37L1 in renal tubular epithelial cells enhances Na^+^/H^+^ exchanger isoform 3 (NHE3) activity, thereby promoting natriuresis and diuresis. This regulation potentially involves cAMP dynamics and PI3K/AKT/mTOR signaling (Zheng et al., 2019; Armando et al., 2022). Notably, GPCR-NHE3 crosstalk establishes a novel paradigm for fluid-electrolyte homeostasis via coordinated intra- and extracellular pH/ion balance. For example, OGR1 inhibits NHE3 activity to mediate renal calcium excretion (Imenez Silva et al., 2020), whereas GPRC5C elevates its activity to regulate systemic pH (Rajkumar et al., 2018). Furthermore, renal perfusion-sodium excretion equilibrium crucially maintains blood pressure stability, with key contributions from Dopamine receptors (DRs) (Yang J. et al., 2021), prostaglandin receptors (EPRs) (Wang et al., 2022), and Angiotensin Receptors (ATRs) (Colafella et al., 2016).

**TABLE 2:** GPCR-Mediated Mechanisms in Renal physiology and pathology.

GPCR and kidney	GPCRs	Renal region	Model	Mechanisms	Outcomes	Ref.
GPCR-mediated-renal physiological functions	GPR126	Tubular epithelial cells	Zebrafish, Mice, and humans	Regulation of Ca^2+^ homeostasis and modulation of pH	Renal progenitor cell differentiation and kidney development	Cazorla-Vázquez et al. (2023)
	LGR4	Renal cells	Mice and Humans	Initiation of Wnt-driven developmental processes	Regulates the formation of the kidney epithelium	Filipowska et al. (2022)
	GPRC5C	Proximal tubular cells	Mice	Enhancement of NHE3 activity	Regulates pH homeostasis	Rajkumar and Pluznick (2018)
	CaSR	Proximal tubular cells		Ca^2+^ homeostasis	Regulates renal fluid, electrolyte, urinary acidification, and blood pressure	Riccardi and Brown (2010)
	SCTR	Medullary and proximal tubular cells	Various vertebrates	Cyclic adenosine monophosphate-induced trafficking of AQP2 or indirectly through the regulation of other hormones		Bai et al. (2017)
	TGR5	Collecting tubules	Mice	Regulates AQP2		Li et al. (2018)
	V2R	Medullary and cortical collecting ducts		Modifies the trafficking of AQP2, ENaC, and urea transporters		Juul et al. (2014)
	P2Y2R	Proximal tubule and Henle’s loop	Rats and mice	Regulates ATP/uridine triphosphate/P2Y2R system and inhibits ENaC activity, increasing renal sodium excretion		Soares et al. (2023)
GPCR-mediated renal pathological processes	PAR2	Tubular cells	RF mice	MAPK-NF-κB signaling	Increases inflammatory responses and EMT	Ha et al. (2022)
	AT1R	Tubular cells	RF rats	Regulates β-arrestin-2	Regulates extracellular matrix synthesis	Wang et al. (2017a)
	C5aR1	Podocytes	Lupus-prone mice	Enhances dynamin-related protein 1-mediated mitochondrial fission	Promotes podocyte injury	Ye et al. (2024)
	GPR97	Proximal and distal tubules	Hypertensive nephropathy mice and AKI mice	Facilitates TGF-β signaling and attenuates the expression of semaphorin 3A	Contributes to hypertension-associated tubulointerstitial fibrosis and exacerbates AKI	Wu et al. (2023), Fang et al. (2018)
	PAR1	Tubular cells	AKI-to-CKD mice	Modulates TGF-β/Smad, NF-κB, and extracellular signal-regulated kinase/MAPK pathways; M1- and M2-polarized macrophages	Promotes renal tubular injury, inflammatory responses and fibrosis	Lok et al. (2023)
	CXCR4	Tubular cells	RF mice	Regulates TGF-β1 and BMP7 pathways; p38/MAPK and PI3K/AKT/mTOR signaling		Yuan et al. (2015), Cao et al. (2022)
	LPAR1/LPAR2	Proximal tubular cells	AKI-CKD rat	Reduces TGF-β-Smad2/3, TGF-β-GSK-3β signaling		Geng et al. (2021)
	CB2R	Tubular epithelial cells	RF mice	Mediates fibroblast and macrophage activation		Zhou et al. (2018)
	GRPR	Renal tubular epithelial cells	Hyperuricemic nephropathy mice	Suppresses the ABCG2/PDZK1 and increases TGF-β/Smad3 levels by activating the NF-κB pathway		Sun et al. (2023)

GPCRs, are primarily involved in renal physiological functions, including renal development, regulation of water-salt metabolism, pH homeostasis, and blood pressure balance, as well as pathological processes such as renal inflammation, mesenchymal transition, ECM, accumulation, and RF. NHE3: Na+/H+ exchanger 3; AQP2: aquaporin 2; ENaC: epithelial Na + channels; ATP: adenosine triphosphate; MAPK: mitogen-activated protein kinase; NF-κB: nuclear factor kappa-light-chain-enhancer of activated B cells; EMT: Epithelial-Mesenchymal Transition; TGF-β: transforming growth factor-β; AKI: acute kidney injury; BMP7: bone morphogenetic protein 7; PI3K: Phosphatidylinositol 3-Kinase; AKT: Protein Kinase B; mTOR: mammalian target of rapamycin; GSK-3β: glycogen synthase kinase 3 beta; ABCG2: ATP-Binding Cassette Subfamily G Member 2; PDZK1: PDZ, Domain-Containing 1.

Meanwhile, the GPCR superfamily orchestrates pivotal pathological processes in renal diseases, including inflammatory cascades, immune dysregulation, fluid-electrolyte imbalances, and RF (Lv et al., 2024). Inflammation serves as the primary instigator of renal injury, wherein complement C5aR activation drives pathogenesis in inflammatory nephropathies such as lupus nephritis (Ye et al., 2024), ANCA-associated vasculitis (Xiao et al., 2014), and acute pyelonephritis (Li et al., 2017). Autoimmune mechanisms further characterize renal pathology, with chemokine receptors (CCRs) orchestrating leukocyte trafficking and tissue infiltration (Hamdan and Robinson, 2021). Notably, CXCR3-dependent immune cell crosstalk represents an emerging therapeutic target (Yoshikawa et al., 2023). In contrast to normal homeostatic functions, AT1R (Dalman and Coleman, 2023), V2R (Bankir et al., 2010), and ETRs (Hunter et al., 2017) promotes sodium-water retention and hypertensive nephropathy (HN). RF, a hallmark pathological endpoint of progressive CKD, is orchestrated by GPCRs at multiple regulatory tiers. For instance, Prostaglandin E2 (EP2) engages four distinct EPR subtypes to stimulate diverse intracellular signaling cascades (Mutsaers and Nørregaard, 2022). LPA activates six GPCR subtypes that drive immune cell recruitment and sustain profibrotic mediator production (Park and Miller, 2017). Emerging evidence further elucidates the contributions of ORs (Motahharynia et al., 2022), GPCR-Gβγ complexes (Kamal et al., 2017), and GPCR-β-arrestin-biased pathways (Gu et al., 2015) in RF pathogenesis, collectively unveiling viable therapeutic strategies to reverse fibrosis. In summary, GPCRs exhibit profound dualistic involvement in renal physiology and pathobiology, positioning them as high-priority therapeutic targets for innovative renal disease interventions.

## 4 Key GPCRs in RF

### 4.1 Endothelin receptors

Accumulating evidence implicates ETRs are involved in the pathological changes of RF. Typically, endothelin-1 (ET-1) initiates the Gq/G11 signaling cascade to trigger downstream Ca^2+^ mobilization, thereby activating both ETAR and ETBR. Interestingly, ligand-stimulated ETAR and ETBR exhibit functionally antagonistic roles in renal pathophysiology (Mazzuca and Khalil, 2012). ETBR activation causes vasodilation and clears ET-1, conferring renoprotective effects, whereas ETAR activation primarily exerts vasoconstrictive effects (Martínez-Díaz et al., 2023). This vasoconstrictive response correlates with increased renal vascular resistance, cortical/medullary vasoconstriction, mesangial cell contraction, and stimulated ECM production (Neuhofer and Pittrow, 2006). Notably, compared to other organs, renal ETRs exhibit heightened sensitivity to ET-1. Critically, ETRs are expressed throughout the kidney, with particularly high levels of ET-1 and ETAR in podocytes and mesangial cells (Anguiano et al., 2015) – cell types recognized as major precursors of fibrogenic fibroblasts (Roccatello et al., 2024). Consequently, ETAR antagonism represents a strategic therapeutic target for RF suppression by effectively inhibiting renal fibroblast proliferation, reducing ECM deposition and antagonizing pro-fibrotic mediators such as ET-1, TGF-β, angiotensin II, and aldosterone (Kohan et al., 2023). While initial monotherapies revealed paradoxical fluid retention risks (Schinzari et al., 2024), contemporary regimens combining ETAR antagonists with ATR blockers or SGLT2i demonstrate optimized efficacy in reducing albuminuria while mitigating hydrostatic complications (Rovin et al., 2023; Heerspink et al., 2023). FDA-approved dual-targeting agents sparsentan (ETAR/AT1R antagonist) and aprocitentan(ETAR/ETBR antagonist) exemplify this synergistic therapeutic approa-ch (Smeijer et al., 2025). Collectively, ETRs signaling constitutes a mechanistically validated axis for targeted RF intervention.

### 4.2 Angiotensin receptors

Recently, Renin-angiotensin-aldosterone system (RAAS) inhibitors now constitute the foundational pharmacotherapy for CKD. wherein ATR subtypes play pivotal roles and their anti-fibrotic properties have garnered increasing scientific attention (AlQudah et al., 2020). Angiotensin II stimulation diversely engages AT1R through Gq/11, Gi/o, G12/13, and β-arrestin pathways to orchestrate pro-fibrotic cascades (Tóth et al., 2018), while AT2R signals through Gi cascades to exert anti-fibrotic effects (Azushima et al., 2020). Mechanistically, AT1R activation promotes vasoconstriction, inflammatory responses, oxidative stress, and fibrogenesis, whereas AT2R activation partially antagonizes AT1R-mediated pathological processes (Forrester et al., 2018). This functional opposition is exemplified by β-arrestin-biased AT1R signaling, which elicits rapid intracellular Ca^2+^ transients in podocytes—accelerating podocyte detachment and glomerulosclerosis (Semenikhina et al., 2023). Conversely, AT2R activation confers renoprotection against fibrosis by modulating Ca^2+^ handling dynamics (Wang et al., 2017b). In summary, although clinical applications targeting angiotensin receptors are well-established, developing innovative ligands for dual receptor modulation and elucidating their spatiotemporal signaling dynamics constitute active investigative frontiers in nephrology (Chow et al., 2019).

### 4.3 Chemokine receptors

Chemokines represent a class of chemotactic cytokines classified into four structural subtypes: XCL, CXCL, CCL, and CX3CL. Their cognate receptors similarly comprise four families: XCR, CXCR, CCR, and CX3CR (Wu F. et al., 2020). These receptor systems critically regulate cellular migration, proliferation, and adhesion dynamics, thereby modulating renal disease progression and regression (Lai and Mueller, 2021). Typically, chemokine coupling to G proteins activates both Gi and Gq pathways, mobilizing secondary messengers including cAMP and Ca^2+^ that mediate heterogeneous biological outcomes. Distinct CCR mediate heterogeneous biological effects through these cascades (Legler and Thelen, 2018; Zweemer et al., 2014). After kidney injury, activated inflammatory cells release chemokines that bind specifically to cognate receptors on immune cells, and orchestrate inflammatory cell recruitment to injury sites, thereby accelerating RF (Yoshikawa et al., 2023; Wu F. et al., 2020). CCR2, a specific pro-fibrotic gene in CKD (Fu et al., 2024), recruits Vδ1 T cells infiltration into renal parenchyma, promoting interstitial fibrosis in IgA nephropathy (Deng et al., 2023). Notably, CCR2 also exerts fibrogenic effects in renal resident cells, including podocytes, independent of immune cell recruitment, indicating that cell-specific CCR2 targeting may offer improved therapeutic precision (You et al., 2017). Furthermore, substantial evidence demonstrates that chemokine axes—including CXCL12/CXCR4 (Chen et al., 2024), CCL20/CCR6 (Zhu et al., 2024), and CXCL5/CXCR2 (Chang et al., 2024)—drive RF progression. Conversely, atypical chemokine receptors (ACKRs) exert counter-regulatory effects in RF, ACKR2 attenuates fibrosis by scavenging CCL2, thereby limiting immune cell and fibroblast infiltration into the interstitium. ACKR2 deficiency, however, exacerbates RF (Eller and Rosenkranz, 2018; Lux et al., 2019). In summary, the CCR network represents a druggable target system for intercepting multifactorial fibrogenic pathways in renal disease.

### 4.4 Adenosine receptors

Extracellular adenosine accumulates pathognomonically during chronic inflammation and hypoxia, with sustained elevations stimulating downstream signaling through four GPCRs: A1AR; A2AR; A2BR; and A3AR. These GPCRs exhibit differential G-protein coupling, Typically, adenosine stimulation induces A1AR and A3AR preferentially engage Gi pathways, while A2AR and A2BR signal through Gs pathways, collectively mediating downstream cAMP signaling transduction (Borea et al., 2018). In RF, A1AR and A2AR activation attenuates EMT/EndMT and ECM accumulation, exerting renoprotective effects (Tian et al., 2021; Chen et al., 2019). Conversely, A2BR and A3AR activation drive profibrotic pathways to accelerate disease progression (Dai et al., 2011; Yu et al., 2019). Notably, receptor functions demonstrate anatomical and mechanistic specialization, A1AR modulates hemodynamic homeostasis through its association with afferent arteriolar vasoconstriction, whereas A3AR primarily underlies metabolic disorder-driven renal injury. Conversely, A2AR and A2BR exhibit stronger associations with direct profibrotic pathways—specifically mesenchymal transition and ECM dysregulation (Dorotea et al., 2018; Li et al., 2012; Roberts et al., 2014). Thus, the AR family exerts complex and context-dependent effects on RF pathogenesis, mediated through GPCR signaling pathways.

### 4.5 Other GPCRs

In addition to the aforementioned GPCRs involved in RF, multiple additional GPCR families—including LPARs (Lee et al., 2019), protease-activated receptors (PARs) (Bagang et al., 2023), cannabinoid receptors (CBRs) (Barutta et al., 2018), and prostaglandin E receptors (EPRs) (Nasrallah et al., 2014)—contribute to fibrogenesis through distinct pathological mechanisms. Significantly, orphan GPCRs (oGPCRs) —defined by unidentified endogenous ligands—have emerged as critical microenvironmental sensors (Rajkumar and Pluznick, 2017). Members of the retinoic acid-inducible GPRC5 subfamily exhibit cell-type-specific pathophysiological roles, Podocyte-localized GPRC5A attenuates fibrosis by suppressing TGF-β-mediated glomerular basement membrane thickening and mesangial hyperplasia (Ma et al., 2018); GPRC5B conversely exacerbates fibrogenesis via NF-κB-driven podocyte inflammation (Zambrano et al., 2019); Tubular GPRC5C primarily modulates acid-base homeostasis (Rajkumar et al., 2018). Additionally, orphan receptor GPR176 demonstrates fibroblast-specific enrichment where it promotes fibroblast activation through TGF-β-independent pathways (Okamoto et al., 2024), positioning orphan receptor as compelling therapeutic targets. Furthermore, Emerging evidence further implicates ectopically expressed ORs in renal pathology (Wu C. et al., 2024), with Olfr433 showing specific enrichment in injury-responsive renal macrophages—suggesting direct involvement in fibrotic cascades (Motahharynia et al., 2022). Collectively, these findings substantiate the multidimensional regulatory architecture of GPCRs networks in RF pathogenesis and reveal novel druggable nodes for anti-fibrotic intervention.

## 5 GPCRs are involved in the pathological phenotypic transition in renal fibrosis

### 5.1 Early infiltration of inflammatory cells and production of pro-fibrotic factors

**FIGURE 2:**
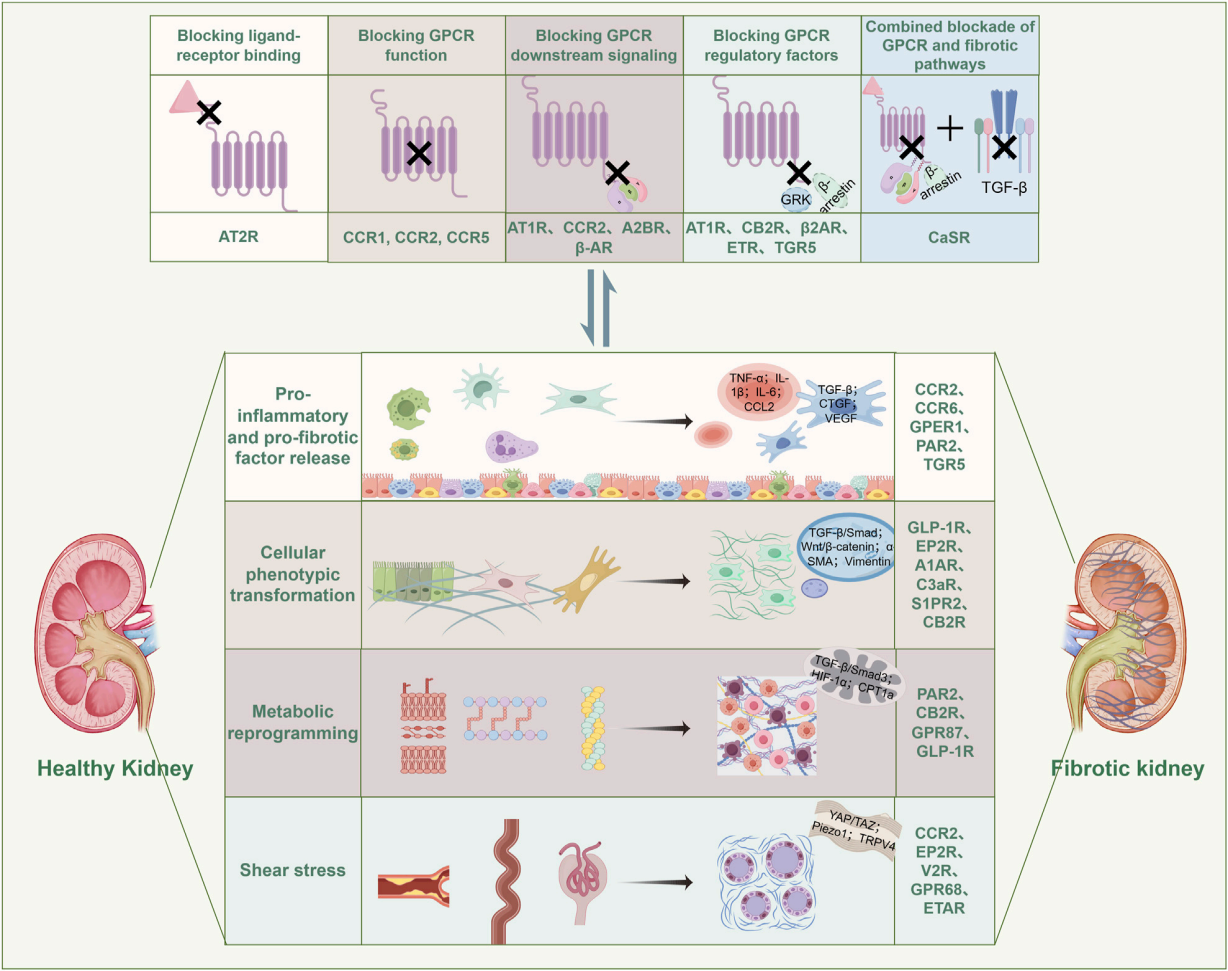
GPCR-mediated signal transduction and pathological alterations in RF engage in a reciprocal interplay. Specifically, GPCRs are involved in the release of pro-inflammatory and pro-fibrotic factors, cellular phenotypic transitions, metabolic reprogramming, and mechanical stress-induced injury during RF progression. Conversely, the activity of GPCRs is also modulated by the pathological changes inherent to RF. GRK: G protein-Coupled Receptor Kinase; TGF-β: Transforming Growth Factor-β; TNF-α: Tumor Necrosis Factor-α; IL-1β: Interleukin-1β; IL-6: Interleukin-6; CCL2: C-C Motif Chemokine Ligand 2; CTGF: Connective Tissue Growth Factor; VEGF: Vascular Endothelial Growth Factor; α-SMA: α-Smooth Muscle Actin; HIF-1α: Hypoxia-Inducible Factor-1α; YAP/TAZ: Yes-Associated Protein/Transcriptional Co-Activator with PDZ-Binding Motif; Piezo1: Piezo Type Mechanosensitive Ion Channel Component 1; TRPV4: Transient Receptor Potential Vanilloid 4.

In the early stages of renal injury, GPCRs critically mediate inflammatory cell infiltration and pro-fibrotic factor release, serving as pivotal initiators of RF progression (Meng, 2019) (Figure 2) (Table 3). This pathogenic cascade is characterized by damage-associated molecular patterns (DAMPs) activating pattern recognition receptors post-injury, triggering immune cell recruitment and polarization that amplify fibrogenic signaling networks (Zhou et al., 2020; Anders and Schaefer, 2014). CCRs constitute essential molecular conduits in this process (Zhou et al., 2020): CXCL16 functions as a scavenger receptor binding oxidized LDL (oxLDL), exhibiting tubular epithelial upregulation that activates CXCR6^+^ fibroblasts to potentiate tubular injury (Korbecki et al., 2021); concurrently, CCL2 induces ACKR2 expression in renal interstitial lymphatic endothelial cells, attenuating CD4^+^ T-cell and mononuclear phagocyte infiltration while suppressing inflammatory cascades (Bideak et al., 2018). Additional receptors including CCR6 (Zhu et al., 2024), GPER1 (Xie et al., 2023), and PAR-1 (Lok et al., 2023) regulate macrophage infiltration and M0-to-M1/M2 phenotypic polarization. Critically, GPR120 agonism in in vitro-programmed peritoneal macrophages sustains the M2 phenotype, thereby inhibiting EMT and conferring renoprotection (Wang et al., 2019). These findings collectively indicate that early-phase reprogramming of inflammatory cells represents a strategic intervention to decelerate inflammation-fibrosis transition.

**TABLE 3:** The role of GPCRs in phenotypic transformation in RF.

Pathological phenotype	GPCR	Stimulus	Model	Mechanisms	Marker	Ref.
Early infiltration of inflammatory cells and production of pro-fibrotic factors	PAFR	PAFR-KO	UUO mice	Regulates the renal pro-inflammatory environment	TNF-α, IL-1β, IL-6, MCP-1 *↓*	Correa-Costa et al. (2014)
	CCR2	CCR2 antagonist RS102895	IRI mice	Regulates MCP-1/CCR2 signaling	TNF-α*,*PDGFβ*,* TGF-β, *Nos2 ↓*	Xu et al. (2019)
	GPER1	GPER1 agonist G-1	UUO mice	Inhibits M1 and M2 macrophage activation	CD86, NLRP3*,* TNF-α*,* IL-1β*, Nos2 ↓*	Xie et al. (2023)
	PAR2	PAR2-KO	Adenine diet-induced RF mice	Increases PAR2/MAPK signaling	Ccl2, Ccl3, Ccl5, Ccl7, TNF-α, IL*-*6, and IL-1β *↓*	Ha et al. (2022)
	DR		Mice with AKI,CKD, or RF	Regulates NLRP3/apoptosis-associated speck-like protein containing a CARD/caspase-1/IL-1β/IL-18 pathway	NLRP3, IL-1β, IL-18 *↓*	Henedak et al. (2024)
	GRPR	GRPR knockdown	Hyperuricemic nephropathy mice	Inhibits NF-κB/TGF-β/Smad3 levels	IL-1β, NF-κB *↓*	Sun et al. (2023)
	TGR5	*Gentiana manshurica Kitagawa*	DN mice	Promotes the interaction of β-arrestin2 with NF-κB inhibitor	NF-κB *↓*	Xiao et al. (2020)
	A2AR	A2AR agonist dexamethasone	LPS induced AKI mice	Inhibits pyroptosis and necroptosis	NLRP3, TGF-β1 *↓*	Sun et al. (2024)
Renal mesenchymal transition	ETBR	ETBR antagonist BQ‐788	Angiotensin II–dependent hypertension rat model	Regulates Rho‐kinase and YAP signaling	E‐cadherin ↑ αSMA *↓*	Seccia et al. (2016)
	A1AR	A1AR antagonist tamsuosin	UUO mice	Regulates A1AR/p38/Smad3 signaling	E‐cadherin ↑ Fibronectin, Vimentin, CTGF, Snail1 *↓*	Ren et al. (2020)
	DOR	DOR agonist UFP-512	TGF-β1-induced NRK-52E	Regulates TGF-β/Smad, Akt, and p38/MAPK signaling	E‐cadherin ↑ Fibronectin, αSMA, Snail *↓*	Luo et al. (2021)
	LGR4	LGR4 siRNA	High-fat diet-induced obesity mice	Regulates Wnt/β-catenin signaling	E‐cadherin ↑ Collagen I, collagen IV *↓*	Su et al. (2021)
	CXCR7	pFlag-CXCR7	IRI and UUO mice	Regulates β-catenin signaling	E-Cadherin ↑ *Collagen I,* αSMA, Fibronectin *↓*	Meng et al. (2024)
	S1PR2	S1PR2 antagonist JTE-013	Differentiated Madin-Darby canine kidney cells	Regulates adherent junction disassembly, β-catenin, and SNAI2 nuclear translocation, and vimentin expression	E-cadherin, Zonula Occludens-1 ↑ Vimentin *↓*	Romero et al. (2023)
	A2AR	Spironolactone upregulated A2AR	Isoprenaline induced renal injury, followed by heart failure	Inhibits EndMT	CD31, VE-cadherin ↑ αSMA, Vimentin *↓*	Chen et al. (2019)
	C3aR	C3aR antagonist	Aristolochic acid nephropathy mice	Reduces inflammation and apoptosis in renal tubular epithelial cells	E-cadherin ↑ αSMA, TGF-β1 *↓*	Ye et al. (2019)
	AT1R/AT2R	Losartan inhibits the RAAS system	Half-nephrectomized B-6 mice and PKSV-PRs	Regulates RAAS/TGF-β/Snail signaling	E-cadherin ↑ TGF-β1, Snail, Fibronectin, αSMA *↓*	Sun et al. (2012)
	GLP-1R	GLP-1R antagonist exendin-3	Monocrotaline induced renal microcirculation lesions rats	Reduce TGF-β 1-associated microcirculatory lesion	von Willebrand factor ↑ *α*SMA, TGF-β1, Smad 3, Snail *↓*	Xu et al. (2018)
Mediating renal fibroblast activation	GPR176	GPR176-KO	UUO mice	Inhibits the TGFβ1/Smads/α-SMA pathway in fibroblasts	TGF-β1, αSMA, Collagen I, Fibronectin 1 *↓*	Okamoto et al. (2024)
	CB1R	CB1R-KO and CB1R antagonist AM6545	UUO mice	Mediates activation of myofibroblasts	αSMA, Collagen IIIa *↓*	Lecru et al. (2015)
	CB2R	CB2R inverse agonist XL-001	UUO-, IRI-, and adriamycin - induced RF mice	Regulates TGFβ1 signaling	TGF-β1, αSMA, Fibronectin, Collagen I *↓*	Zhou et al. (2018)
	A2RB	A2RB antagonist PSB603; MRS1754	NRK-49F; STZ-induced diabetes mellitus rats	Induces an activated fibroblast phenotype; Decreases intraglomerular macrophage infiltration and macrophage–myofibroblast transition	αSMA, IL-6, TGF-β, CTGF, Collagen Ia, and Fibronectin *↓*	Torres et al. (2020)
	V2R	V2R agonist	*Pkd1*-KO mice	Regulates V2R-YAP-CCN2 cell signaling	αSMA, Collagen Ia, Collagen IIIa *↓*	Dwivedi et al. (2020)
	CXCR6	CXCL16-KO(CXCR6 ligand)	UUO mice	Inhibits the recruitment of fibroblast precursors	αSMA, Collagen I, Fibronectin *↓*	Chen et al. (2011)
Modulating extracellular matrix accumulation	A1AR	A1AR agonist	STZ-induced diabetes mice	Regulates the integrity of the tubular microenvironment	Collagen I, III, and IV, TGF-β*,* αSMA, Vimentin *↓*	Tian et al. (2021)
	DR1	DR1 agonist SKF38393	STZ-induced diabetes mice	Downregulates the ERK1/2 signaling	MMP9 ↑ αSMA, Collagen I *↓*	Li et al. (2022c)
	AT1R	Candesartan (AT1R blocker)	NRK-49F	Regulates AT1R-β-arrestin-2-ERK1/2 signaling	Collagen I, Fibronectin *↓*	Wang et al. (2017a)
	EP1R	Mesangial cells from EP1R-deficient mice	TGF-β1-induced mesangial cells	Regulates the reinforcement of ERK phosphorylation	Collagen I, Fibronectin *↓*	Chen et al. (2015)
	CX3CR1	CX3CR1-KO	STZ-induced DN mice	Activates ROS and MAPKs	TGF-β1, Fibronectin, Collagen IV α1*,* Fractional mesangial area *↓*	Song et al. (2013)
	S1PR2	Berberine	STZ-induced diabetes mice	Regulates NF-κB activation	Fibronectin *↓*	Huang et al. (2012)

GPCRs, inhibit RF, by interfering with pathological phenotypic transitions, including the early release of pro-inflammatory and pro-fibrotic factors, modulation of mesenchymal transition, activation of fibroblasts, and accumulation of extracellular matrix. KO: knockout; UUO: unilateral ureteral obstruction; TNF-α: Tumor necrosis factor-alpha; IL-1β*:* interleukin-1, beta; IL-6:interleukin-6; MCP-1: monocyte chemoattractant protein-1; IRI: Ischemia-reperfusion injury; PDGFβ: platelet-derived growth factor beta; TGF-β: *transforming growth factor-beta*; *Nos2:* nitric oxide synthase 2; CD86: *Cluster of differentiation 86*; *Nlrp3: NLR, family pyrin domain-containing 3; CCL:* C-C motif chemokine ligand; MAPK: Mitogen-Activated Protein Kinase; AKI: acute kidney injury, CKD: chronic renal injury; CKD: chronic kidney disease; NF-κB: nuclear factor kappa-light-chain-enhancer of activated B cells; DN: diabetic nephropathy; LPS: lipopolysaccharide; HN: hyperuricemic nephropathy; YAP: Yes-associated protein; αSMA: α-Smooth muscle actin; CTGF: connective tissue growth factor; EndMT: Endothelial-to-Mesenchymal Transition; RAAS: renin-angiotensin-aldosterone system; STZ: streptozotocin; CTGF: connective tissue growth factor; CCN2: connective tissue growth factor; ERK1/2: extracellular signal-regulated kinase 1/2; MMP9: Matrix metalloproteinase 9; DN: diabetic nephropathy; ROS: reactive oxygen species.

### 5.2 GPCR is involved in cellular crosstalk and phenotypic transformation in RF

Persistent research has established that cellular phenotypic transitions following renal injury constitute a central mechanism in RF pathogenesis (Liu, 2011). Within this process, apoptosis/necrosis of renal tubular epithelial cells, endothelial cell injury, and immune cell infiltration converge to activate matrix-producing myofibroblasts, which directly drives ECM accumulation and fibrotic phenotypic remodeling (Huang et al., 2023). Substantial evidence implicates GPCRs in orchestrating multiple phenotypic transitions during RF (Tang et al., 2025). For instance, during early disease stages, CCR2-expressing monocytes exhibit heightened differentiation into pro-inflammatory macrophages. subsequently driving macrophage-to-myofibroblast transdifferentiation that accelerates fibrogenesis (Xu et al., 2019; Braga et al., 2018). Concurrently, epithelial and endothelial cells undergo loss of polarity, transitioning from tightly adherent, organized morphologies to detached spindle-shaped structures that promote mesenchymal transition and fibrogenesis (Jacobs et al., 2024; Fintha et al., 2019). Pharmacological blockade of PAR-1 (Saifi et al., 2021) and A1AR (Ren et al., 2020) or EP2R (Jensen et al., 2019) activation effectively downregulates mesenchymal markers to attenuate RF. Notably, GPCR expression profiling in renal fibroblasts reveals significant enrichment of S1PR3 and A2AR/A2BR subtypes (Kaur et al., 2023), with sphingosine-1-phosphate (S1P) or its analogs directly stimulating fibroblast activation (Shiohira et al., 2013), while A2BR activation has been definitively demonstrated to drive macrophage-to-myofibroblast conversion, further amplifying fibrotic cascades (Torres et al., 2020). Thus, GPCR-mediated control over cellular phenotypic transitions constitutes a defining pathomechanism in RF, positioning these receptors as privileged therapeutic targets for intercepting fibrotic progression.

### 5.3 GPCR is involved in metabolic reprogramming in RF

Metabolic reprogramming—manifested by pathological remodeling of fatty acid β-oxidation (FAO), dysregulated aerobic glycolysis, mitochondrial insufficiency, and inflammatory infiltration, This reprogramming sustains heightened bioenergetic demands during fibrogenesis through altered substrate utilization (Zhu et al., 2021; Miguel et al., 2025; Zhu Z. et al., 2022). Substantial evidence establishes GPCRs as master regulators of metabolic flux in RF, particularly via the Gα12/13 signaling (Yang et al., 2020); for instance, PAR2 and CB2R activation induce tubular epithelial cell senescence and lipid droplet accumulation, impairing mitochondrial β-oxidation capacity (Ha et al., 2024; Zhou et al., 2024), while GPR87 accelerates glycolysis and mitochondrial damage, promoting ECM deposition (Cui et al., 2022). Beyond direct metabolic regulation, GLP-1R agonists normalizes lipidomic profiles and mitochondrial metabolites (acyl-carnitines, cholesterol, succinate), conferring renoprotection (Wang et al., 2018). Conversely, microbiota-derived metabolites serve as endogenous GPCR ligands (Rhee, 2018), exemplified by butyrate—GPR109a axis activation preserving podocyte integrity against glomerular basement membrane injury (Felizardo et al., 2019). Additionally, injured renal cells exhibit secretory dysfunction, the secretome of renal vascular endothelial cells serves as pivotal regulators of fibroblast activation (Lipphardt et al., 2017), exemplified by α2A-AR-driven β-arrestin2 signaling that promotes tubular senescence and pro-inflammatory cytokine secretion, thereby driving fibroblast activation and propagating RF (Li et al., 2022). Collectively, GPCR-mediated governance of metabolic reprogramming pathways represents a frontier in contemporary RF pathobiology research.

### 5.4 GPCRs orchestrate shear stress-induced injury in RF

Contemporary research has delineated shear stress—a fundamental biomechanical force—as a key driver of fibrotic pathogenesis through mechanosensation-signal transduction-epigenetic remodeling cascades (Long et al., 2022), with GPCRs serving as primary mechanosensors and signaling hubs that represent promising therapeutic targets for RF induced by tubular dilation, obstruction, and hyperfiltration (Xiao et al., 2023). The pathophysiological impact manifests through mechanosensitive injury across multiple renal cell types, exemplified by the shear-sensitive ion channel Piezo1 modulating CCR2-mediated macrophage inflammation to suppress mesenchymal transition and RF progression (He et al., 2022), while Yes - associated protein (YAP) —a transcriptional co-activator central to mechanotransduction (Panciera et al., 2017)—participates in myofibroblast activation via the V2R-YAP signaling axis (Jamadar et al., 2022), and EP2R functions as a pathological shear stress sensor in podocytes, directly driving cytoskeletal destabilization and detachment (Srivastava et al., 2018). Furthermore, multiple Gq/11-coupled GPCRs, including GPR68, ETAR, V1AR, and S1PR, demonstrate mechanosensory capabilities, though their precise mechanistic underpinnings warrant further investigation (Xiao et al., 2023). Collectively, GPCRs constitute pivotal mechanotransductive regulators of shear stress-induced renal parenchymal damage, presenting profound pathobiological significance and compelling therapeutic relevance for targeted intervention.

## 6 Challenges and prospects of GPCR target development in RF

Notwithstanding the preeminent status of GPCRs as the most therapeutically exploited target class, their translational deployment against fibrotic disorders remains incipient (Tang et al., 2025; Rieder et al., 2025). This therapeutic inertia predominantly arises from the intricately orchestrated, multifactorial pathoetiology of organ fibrosis, characterized by dynamic oscillations between inflammatory and profibrotic signaling cascades (Abbad et al., 2025). Mononodal pharmacotherapeutic interventions targeting singular nodal points are frequently subverted by compensatory pathway rewiring—a phenomenon starkly evidenced by terminated clinical trials targeting canonical profibrotic networks (e.g., TGF-β, PI3K/mTOR, JAK/STAT) (Di et al., 2025; Zhao et al., 2022). Concomitantly, extant *in vitro* and *in vivo* fibrosis models exhibit limited recapitulation of the human pathophysiological niche, thereby compromising translational fidelity (Addario et al., 2025). Furthermore, the pathologically remodeled ECM in RF imposes steric hindrance that severely restricts lesional drug bioavailability (Xu et al., 2021). Therefore, overcoming the bottlenecks in targeted GPCR intervention for organ fibrosis is of crucial importance.

Despite these challenges, combining computational and experimental tools is driving significant progress. Innovations in 3D microphysiological systems—encompassing organ-on-chip platforms with multicellular co-cultures, vascularized bioprinted constructs, and patient-derived organoids—are progressively standardizing human-relevant fibrotic pathomimetics (Addario et al., 2025; Sacchi et al., 2020; Miyoshi et al., 2020). Parallel breakthroughs in nanotherapeutic delivery—including lipid-encapsulated GPCR ligands, renal-compartment-specific targeting moieties, and pathology-responsive nanovehicles—are circumventing biodistribution barriers (Oroojalian et al., 2020). In GPCR drug discovery, AI-driven compound design and biased ligand development are reaching maturity (Zhang et al., 2024; Yang D. et al., 2021), GPCR-targeted candidates now make up over 60% of receptor-focused clinical pipelines for fibrosis. Key examples include clinical trials targeting S1PR (e.g., Fingolimod), CCR2 (e.g., DMX-200), and GLP-1R (e.g., Exenatide) epitomize this mechanistic momentum (Abbad et al., 2025). Collectively, the precision targeting of GPCR signaling nodes harbors exceptional potential for intercepting the fibrotic cascade at its evolutionary nexus.

## 7 Conclusion

Given the persistent high disease burden and suboptimal therapeutic outcomes in RF, convergent preclinical and clinical evidence has validated the therapeutic tractability of GPCRs. This review delineates the pathophysiological primacy of key GPCR families—notably endothelin, angiotensin, chemokine, and adenosine receptors—in orchestrating RF progression through multimodal regulation spanning inflammatory/fibrogenic cascade initiation, maladaptive cellular phenotypic transitions, metabolomic reprogramming, and mechanotransductive injury responses. Collectively, GPCRs emerge as supramolecular signaling hubs whose precision modulation holds exceptional promise for next-generation anti-fibrotic therapeutics.

## Glossary

RF: Renal fibrosis
GPCRs: G protein-coupled receptors
CKD: Chronic kidney disease
ESRD: End-stage renal disease
EMT: Epithelial - Mesenchymal Transition
EndMT: Endothelial - Mesenchymal Transition
ECM: Extracellular matrix
ACEIs: Angiotensin-converting enzyme inhibitors
ARBs: Angiotensin II receptor blockers
MRAs: Mineralocorticoid receptor antagonists
SGLT-2i: Sodium-glucose co-transporter 2 inhibitors
ETR: Endothelin receptor
TGF-β: Transforming growth factor-β
GDP: Guanosine diphosphate
GTP: Guanosine triphosphate
AC: Adenylate cyclase
PLCβ: Phospholipase C-β
PIP2: Phosphatidylinositol-4,5-bisphosphate
IP3: Inositol 1,4,5-trisphosphate
DAG: Diacylglycerol
PKC: Protein kinase C
RGS: Regulator of G protein Signaling
GAP: GTPase-activating protein
ncRNAs: non-coding RNAs
aGPCRs: Adhesion GPCRs
DN: Diabetic nephropathy
GBM: Glomerular basement membrane
ARs: adrenergic receptors
LPARs: lysophosphatidic acid receptors
ETRs: Endothelin receptors
ORs: olfactory receptors
NHE3: Na^+^/H^+^ exchanger isoform 3
DRs: Dopamine receptors
EPRs: Prostaglandin receptors
ATRs: Angiotensin Receptors
CCRs: chemokine receptors
HN: hypertensive nephropathy
EP2: Prostaglandin E2
ET-1: Endothelin-1
RAAS: Renin-angiotensin-aldosterone system
ACKRs: Atypical chemokine receptors
PARs: Protease-activated receptors
CBRs: Cannabinoid receptors
DAMPs: damage-associated molecular patterns
oGPCRs: orphan GPCRs
oxLDL: oxidized LDL
S1P: sphingosine-1-phosphate
FAO: fatty acid β-oxidation
YAP: Yes - associated protein
